# Quick and efficient approach to develop genomic resources in orphan species: Application in *Lavandula angustifolia*

**DOI:** 10.1371/journal.pone.0243853

**Published:** 2020-12-11

**Authors:** Berline Fopa Fomeju, Dominique Brunel, Aurélie Bérard, Jean-Baptiste Rivoal, Philippe Gallois, Marie-Christine Le Paslier, Jean-Pierre Bouverat-Bernier

**Affiliations:** 1 ITEIPMAI, Chemillé-en-Anjou, France; 2 US1279 Etude du Polymorphisme des Génomes Végétaux (EPGV), INRA, Université Paris-Saclay, Evry, France; 3 CRIEPPAM, Les Quintrands, Manosque, France; USDA-ARS Southern Regional Research Center, UNITED STATES

## Abstract

Next-Generation Sequencing (NGS) technologies, by reducing the cost and increasing the throughput of sequencing, have opened doors to generate genomic data in a range of previously poorly studied species. In this study, we propose a method for the rapid development of a large-scale molecular resources for orphan species. We studied as an example the true lavender (*Lavandula angustifolia* Mill.), a perennial sub-shrub plant native from the Mediterranean region and whose essential oil have numerous applications in cosmetics, pharmaceuticals, and alternative medicines. The heterozygous clone “Maillette” was used as a reference for DNA and RNA sequencing. We first built a reference Unigene, compound of coding sequences, thanks to *de novo* RNA-seq assembly. Then, we reconstructed the complete genes sequences (with introns and exons) using an Unigene-guided DNA-seq assembly approach. This aimed to maximize the possibilities of finding polymorphism between genetically close individuals despite the lack of a reference genome. Finally, we used these resources for SNP mining within a collection of 16 commercial lavender clones and tested the SNP within the scope of a genetic distance analysis. We obtained a cleaned reference of 8, 030 functionally *in silico* annotated genes. We found 359K polymorphic sites and observed a high SNP frequency (mean of 1 SNP per 90 bp) and a high level of heterozygosity (more than 60% of heterozygous SNP per genotype). On overall, we found similar genetic distances between pairs of clones, which is probably related to the out-crossing nature of the species and the restricted area of cultivation. The proposed method is transferable to other orphan species, requires little bioinformatics resources and can be realized within a year. This is also the first reported large-scale SNP development on *Lavandula angustifolia*. All the genomics resources developed herein are publicly available and provide a rich pool of molecular resources to explore and exploit lavender genetic diversity in breeding programs.

## Introduction

Next-Generation Sequencing (NGS) technologies, by reducing the cost and increasing the throughput of genotyping and sequencing, have opened doors to generate genomic data to a wide range of species that had not yet benefited. In particular, reduced-representation sequencing methods make it possible to develop genomic resources without any prior genomic information, as a reference genome, and thus are particularly suitable to non-model species [1,2]. These methods are either based on the sequencing of DNA fragments after use of restriction enzymes (Restriction-site Associated DNA sequencing (RAD-seq), Genotyping-By-Sequencing (GBS), to cite few), or on the sequencing of the expressed fraction of the genome (exome sequencing, RNA sequencing [RNA-seq]) [3,4]. Among these methods, the *de novo* assembly of RNA-seq has the advantage of allowing the development at reasonable cost of reference sequences, which can subsequently be used as basic genomic resources to start various studies on minor species. Successful examples have been described in chickpea [5] red clover [6] or *Croton tiglium* [7].

One of the limitations of the method is that by assembling mature mRNAs, it is expected that the sequences assembled are restricted to genes’ exons. However, these regions are known to be rather conserved between individuals of a species, or even between different species [8]. In a context of polymorphism discovery, this can present a limit if one works with genetically close individuals. The present study is a proof of concept of a method based on the use of NGS data (from DNA and RNA), that can help to resolve this limit. It is tested for the first time on a species with very limited genomic resources: *Lavandula angustifolia* Mill. The lavender (or true lavender) is a perennial sub-shrub native from the Mediterranean region whom is best known for its essential oils that have numerous applications in perfumes, cosmetics, pharmaceuticals, and alternative medicines [9–11]. The lavender is one major species of the Medicinal and Aromatic Plants (MAP) sector in France: the lavender culture, which includes *L*.*angustifolia* and its hybrid the lavandin *Lavandula x intermedia* (hybrid between *L*. *angustifolia* and L. *latifolia*) represented 48% of the surface area of MAPs in France in 2018 [12].

Initially collected in its natural habitat, the best individuals and the best lavender populations were selected by mass selection and cultivated on plateaus and plains for their essential oil. Nowadays, the new environmental challenges: drought, biotic stresses (mainly lavender decline caused by *Candidatus Phytoplasma solani* [13]*)*, underline the need to select new varieties adapted to these stresses, while keeping good essential oil yields and a quality corresponding to the standards of the market. Traditional breeding may not be sufficient to address those challenges so that it is critical to develop genomic tools helping to improve breeding programs efficiency.

To date, few molecular resources have been developed on *Lavandula angustifolia* and no SNP (Single Nucleotide Polymorphism) markers have been reported. As of June 2019, 340 nucleotide sequences had been deposited in the NCBI’s GenBank database for *Lavandula* species with more than 50% being sequences of *Lavandula angustifolia*. Most of them (~25%) are related to synthesis or regulation of essential oils [14–21]; or are isolated from the chloroplast genome (~30%). Moreover, EST-derived SSR (Simple Sequence Repeat) markers have been recently developed and successfully tested for transferability between *Lavandula* species [22]. In 2018, a genome of *Lavandula angustifolia* have been released [23] and revealed a genome size of 870 megabases. However, that genome was not available in 2017 at the time our analyses were performed, and it is not yet publicly available.

In the present study, our aim was to develop *Lavandula angustifolia* genomic resources and to discover SNP marker to set bases for genomic-assisted breeding in lavender. Firstly, we conducted a regular *de novo* transcriptome assembly from 3 different tissues of the heterozygous lavender clone Maillette. The assembly was cleaned and annotated to get a reference Unigene. Then, we used DNA-seq data from the same clone Maillette to perform a Unigene-guided DNA-seq assembly to recover full-length genes sequences (exons + introns). These genes sequences were subsequently used as reference sequences for SNP discovery from a panel of 16 commercial lavenders and lavandin. Finally, we tested the SNP within the scope of a genetic distance analysis.

The results presented herein offer a solid base for the initiation of population genetics studies, DNA fingerprinting and the development of efficient genomic-assisted selection strategies for *Lavandula angustifolia* and the related species of the *Lamiaceae* family.

## Material and methods

### Plant material

The plant material was selected to be representative of the phenotypic variation (morphological, essential oil constitution) observed in the lavender clones cultivated nowadays (S1 Table). The lavender selection includes two putative geographical origins: Bulgary (2 clones named B6 and B7) and France (remainig clones, mainly from Albion plateau). Almost all the clones were collected from fields of open-pollinated varieties and it was difficult to trace the exact origin and pedigree of each clone. However, some information was provided by the CRIEPPAM (Regional Interprofessional Center for Experimentation in Perfume, Aromatic and Medicinal Plants; Manosque, France) and is indicated in S1 Table. The clone ‘Maillette’ was chosen to construct the reference sequence of lavender because of its large use in the lavender production area. The lavandin ‘Grosso’, a natural sterile interspecific hybrid between *Lavandula angustifolia* and *Lavandula latifolia*, was also included because of its high economic importance in the MAP sector. All clones used are heterozygous, including the reference clone "Maillette". They were all provided by the Technical and Interprofessional Institute of Perfume and Medicinal Plants (ITEIPMAI; Chemillé, France) and CRIEPPAM and were maintained at the ARDEMA (Arid Mountain Research and Development Association) experimental farm (Mévouillon, France).

### RNA and DNA extraction, library construction and sequencing

#### RNA and DNA extraction

To isolate RNA, samples were collected at the end of May. Leaves and root samples were collected from an adult plant of the clone ‘Maillette’ maintained in the field and, as the plant was not flowered, flower buds were collected from an adult plant maintained in a green house. Collected samples were immediately frozen and conserved in liquid nitrogen until use. The samples were grounded in liquid nitrogen. Total RNA was isolated for each tissue sample using Plant RNA Isolation Mini Kit (Agilent, Santa Clara, CA, USA), according to manufacturer’s instructions.

To isolate DNA, leaf samples were collected from 15 lavender clones (including Maillette) and the lavandin ‘Grosso’ at an adult stage, in the field, and immediately frozen and stored in dry ice until use. Total DNA was isolated independently from leaves of the 16 clones according to manufacturer’s recommendations using the NucleoSpin Plant II kit (Macherey-Nagel, Düren, Germany).

After isolation, the yield, purity and integrity of RNA and DNA samples were analyzed using a TapeStation (ADN) or a BioAnalyzer (ARN) (Agilent) device.

#### Library preparation and sequencing

Libraries were prepared independently for each tissue sampled.

After the total RNA was extracted, the cDNA stranded libraries were prepared using the TruSeq stranded mRNA Sample Preparation Kit (Illumina Inc., San Diego, CA, USA) according to manufacturer’s recommendations. The DNA libraries were prepared with the TruSeq DNA PCR-Free Sample Preparation kit (Illumina Inc., San Diego, CA, USA) according manufacturer’s recommendations.

The RNA and DNA libraries were pair-end sequenced on an Illumina HiSeq 2500 (2*150pb).

All sequencing reads were deposited into the Short Read Archive (SRA) of the National Centre for Biotechnology Information (NCBI) and can be accessed under the Bioproject number PRJNA391145.

### Data analysis

#### DNA-seq and RNA-seq trimming

The raw paired-end reads produced following RNA and DNA sequencing were filtered with CLC Genomics Workbench 8.5 (https://www.qiagenbioinformatics.com/), hereafter named CLC, to obtain high-quality cleaned reads. The Illumina adapter sequences, low quality sequences (limit = 0.001), ambiguous nucleotides (no “N” allowed), and short sequences (minimum length = 70 nucleotides) were removed during the trimming process. The tool Trim of CLC uses the limit criterion instead of the commonly used Q criterion for quality trim of raw reads. In practice, setting the limit parameter to 0.001 result in keeping trimmed sequences with an average quality Q > 30.

#### *De novo* assembly of leaf, flower bud and root transcriptome of Maillette

The pipeline used to build our lavender reference Unigene from RNA-seq data is presented on S1 Fig. Each assembly tool has its advantages and its limits and produces different types of bioinformatically derived artefacts. In a recent study, Cerveau and Jackson [24] have shown the interest of surveying the outputs of different assembly tools to generate a high-quality transcriptome. In the present study, we used the popular tools CLC v8.5 and TRINITY v2.1.1 [25,26]. The cleaned paired-end reads from RNA sequencing of leaf, flower bud and root of the lavender clone ‘Maillette’ were pooled and used for the *de novo* transcriptome assemblies. Since the first sequenced read (reads 1) of a pair is often better than the second (reads 2), reads 1 were used to build the primary *De Bruijn* graph and reads 2 were used to resolve bubbles in the graph. The k-mer size values used were 64 bases for CLC and 25 bases for TRINITY (fixed default value).

The quality of the raw assemblies was assessed with TRANSRATE v1.0.2 [27]. TRANSRATE maps paired-end reads back to the raw contigs and calculate metrics (percentage of reads mapping back to contigs in proper pairs, number of contigs with a predicted open reading frame, estimation of fragmented transcripts…), in order to assess how well the assembled contigs are supported by the sequencing data. On this basis, a score is determined for each contig and the set of contigs with best scores are gathered into an “optimized assembly”. TRANSRATE also computes a global score that allows comparing two or more assemblies performed with the same initial dataset but with different tools or settings.

To evaluate the completeness of the assemblies, we performed a BLASTx+ (e-value 1e-20) alignment of the optimized assemblies against SwissProt database (http://www.uniprot.org/) using a script developed by the TRINITY developer’s team (https://github.com/trinityrnaseq/trinityrnaseq/wiki/Counting-Full-Length-Trinity-Transcripts). This analysis allowed to determine (i) the number of unique best blast hit (BBH) that align against contigs and (ii) the percentage of coverage of the hit sequence by the contig.

We finally took advantage of both assemblies to build the reference Unigene. This Unigene is compound of the sequences assembled with TRINITY or CLC that covered more than 70% of their BBH in SwissProt database with more than 30% identity. In case of redundancy within an assembly (*i*.*e*, several contigs matched the same hit in the database), we only kept the longest contig in the Unigene. Likewise, when two sequences built by CLC and TRINITY had the same BBH, we only kept the longest form in the Unigene or, in cases of equality, the one with the highest alignment similarity percentage.

#### *In silico* functional annotation of the reference Unigene

The completeness of the reference Unigene was evaluated with regard to the 1440 plant-specific genes in the Benchmarking Universal Single-Copy Orthologs (BUSCO, Embryophyta data set) [28]. To further describe biological functions related to the Unigene sequences, we ran the Trinotate pipeline (https://trinotate.github.io/) which is a comprehensive annotation suite adapted to *in silico* annotation of *de novo* assembled transcriptome. The analysis includes homology searches (evalue 1e-5) to SwissProt database, protein domain identification using HMMER and Pfam databases, protein signal peptide and transmembrane domain prediction with Signalp and tmHMM servers, and search for Gene Ontology (GO) terms and KEGG pathways [29,30]. Insofar as the ability to annotate sequences based on similarity search against databases depends on the completeness of these databases, we completed the annotations using supplementary databases and tools. The online tools KAAS (http://www.genome.jp/kegg/kaas/) [31] was used with default parameters to decipher associated KEGG pathways by aligning Unigene sequences against the KEGG GENES database. The online tool TRAPID (http://bioinformatics.psb.ugent.be/webtools/trapid/) [32] was also used with default parameters. TRAPID performs open-reading frames detection and similarity searches against implemented databases (OrthoMCL-DB version 5 and PLAZA 2.5). Results were then combined in order to identify coding sequences, assign transcripts to gene families, and generate GO annotation.

#### Iterative targeted assembly of lavender genes

**Fig 1:**
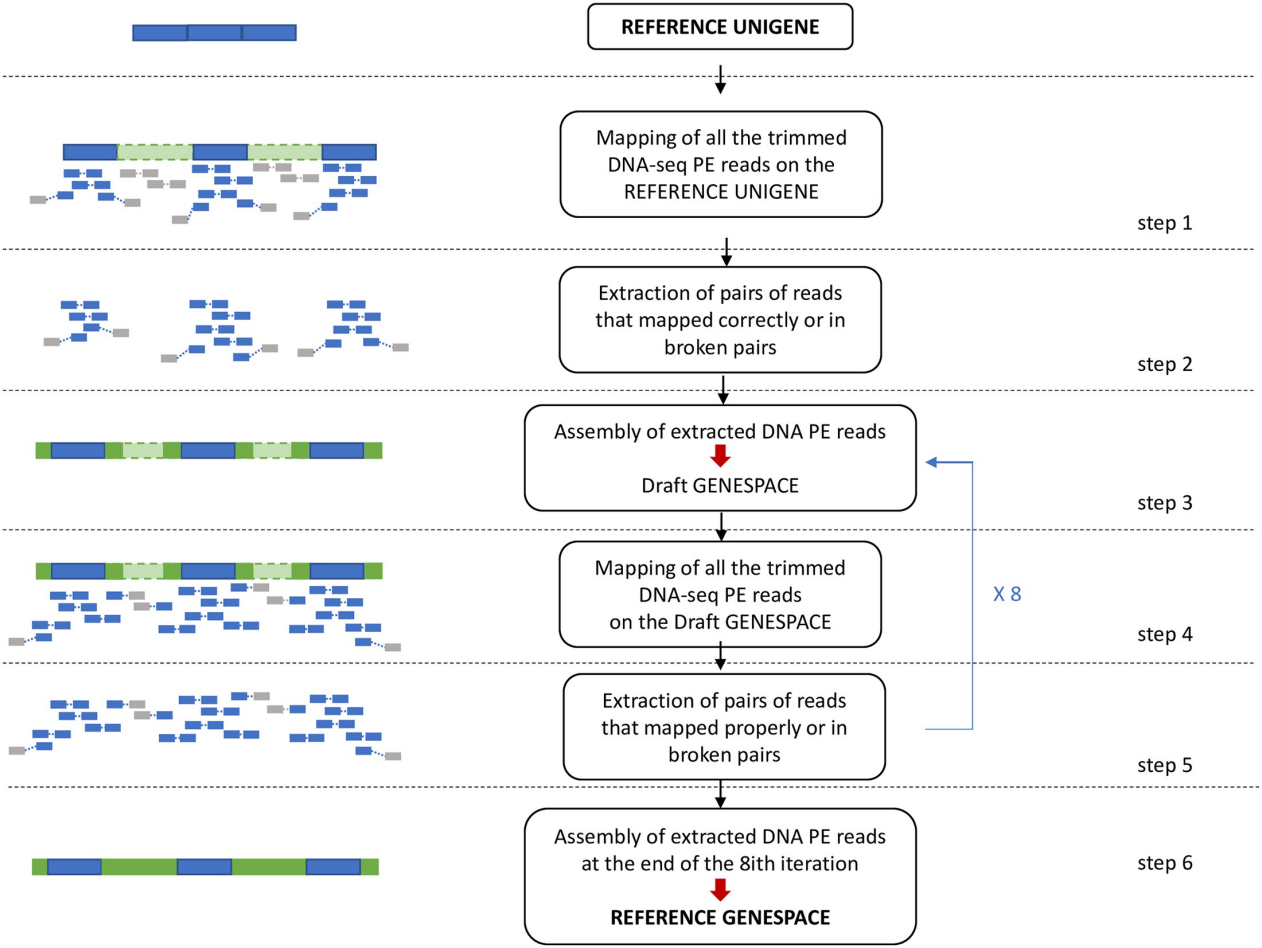
Pipeline for the Unigene-guided DNA assembly for the construction of the reference Genespace. PE: Paired-end.

The Unigene sequences and the reads from the DNA sequencing of the clone ‘Maillette’ were used to perform the iterative mapping and assembly steps to build a set of reference gene sequences (with exons and introns) as described in Aluome *et al* [33] (Fig 1, S1 File), hereafter called Genespace.

Briefly, the first iteration consists in mapping the whole-genome DNA-seq reads on the reference sequences of the Unigene (step 1, Fig 1). Then, the reads that mapped in pairs and broken pairs (*i*.*e*, only one member of the pair mapped) are extracted (step 2, Fig 1) and assembled into *de novo* contigs (step 3, Fig 1). For further iterations, the total reads from DNA sequencing are mapped on the new sequences assembled at the end of the (*i-1*) ^th^ iteration (repetition of steps 3 to 5, Fig 1). We stopped the iterations when the maximum number of sequences from the reference Unigene was re-built in the draft Genespace (in our case, at the 8*th* iteration) (step 6, Fig 1).

CLC Assembly Cell (https://www.qiagenbioinformatics.com/) v 5.0 was used for mapping the reads (CLC_ref_assemble_long tool, step 1) and for extracting the mapped reads (sub_assembly tool, step 2). Mapping was less stringent at the first iteration (step 1, Fig 1: 100% of the read length with a similarity percent of 95%) than for other iterations (step 4, Fig 1: 100% of the read length with a similarity percent of 98%) in order to take into account exons/introns junctions at the first step of mapping. For assembly steps, we used idba_ud v. 1.0.9 software [34]. This tool offers the advantage to use the new sequences assembled at the iteration *i-1* to ‘guide’ the assembly at the iteration *i* (—input-long-read option) and thus allowing to keep the information build at the iteration *i-1* while assembling new sequences at iteration *i*. Parameters used for idba_ud for all iterations were a minimum k-mer size of 25 nucleotides and a maximum k-mer size of 100 nt (—mink 25—maxk 100), with an increment of 5 nucleotides (—step 5) and a similarity value of 1 nucleotides (—similar 1).

Finally, we checked the collinearity and identity (for exons) of the GeneSpace sequences compared to their homolog sequences in the Unigene with a BLASTN alignment (e-value 1e-6).

#### SNP discovery

Filtered DNA-seq paired-end reads of the 15 lavender clones and of the lavandin ‘Grosso’ were mapped against the Genespace sequences with CLC software. To be included in the mapping, at least 95% (90% for ‘Grosso’) of the read (length fraction = 0.95 or 0.90) must be aligned to the reference sequence with at least 90% identity (80% for ‘Grosso’) (similarity fraction = 0.90 or 0.80). Moreover, reads with non-specific matches (*i*.*e*. reads mapping equally well at several alignment positions) were excluded from the mapping.

SNP detection was performed with CLC and GATK v 3.6 [35–37] tools. It has been demonstrated that a significant improvement of SNP calling, in terms of further successful genotyping of the SNP, can be obtained by focusing on SNP discovered by more than one method [38]. According to the initial sequencing depth of the genotypes studied, we applied a maximal depth cutoff of 50X to consider a site for SNP discovery. This maximum cutoff was applied to prevent SNP detection in regions with very high depth of coverage that could correspond to repeated DNA regions.

For variant discovery with CLC, we used the *Fixed Ploidy Level* tool. A diploid model for SNP calling (ploidy level = 2) was selected with a minimum read coverage of 5, a maximum read coverage of 50, a minimum non-reference allele count of 2 with a minimum frequency of 40% (calculated as “number of non-reference alleles at the site”/“total site coverage”). Broken pairs and non-specific reads were not used for SNP detection. The minimum base quality required for the putative SNP and the 5 bases on both sides of the SNP was of 25. For variant detection with GATK, mapping files generated with CLC were exported in *bam* format files to be used in GATK. We used HaplotypeCaller, SelectVariants and VariantFiltration tools included in GATK to perform the analyses. A maximum read coverage of 50 and a minimum per base quality of 30 were required to consider a polymorphic site as a putative variant (since, with GATK, we could not provide a quality parameter for the bases in the vicinity of the called SNP, we defined a quality threshold for the called SNP higher in GATK than in CLC). We then applied SelectVariants and VariantFiltration programs to only select SNP in each vcf files and to apply filters to SNP calling similar to those applied with CLC. Finally, in order to prevent from spurious SNP calling, we applied SelectVariant tool to extract SNP that were concordant (position and genotype information) between CLC and GATK vcf output files to get a cleaned set of putative SNPs.

#### Genetic distances analysis

From the filtered putative SNPs set, we selected SNPs that generated no missing genotype data. The analyses were based on the ‘Population Structure’ workflow from the Grundwald laboratory (https://grunwaldlab.github.io/Population_Genetics_in_R/Pop_Structure.html). The R packages ‘poppr’ version 2.7.1 [39,40], ‘adegenet’ [41,42] and ‘ade4’ [43] were used to calculate absolute genetic distances with ‘Prevosti’s method’ [44] (a method suited to SNP data), to perform a principal component analysis (PCA) and built an UPGMA phylogenetic tree with 1000 bootstraps.

## Results

### RNA-seq and DNA-seq trimming

A total of 169,082,890 (84,541,445 pairs) of RNA paired-end (PE) reads were sequenced reaching 25.5 Gigabases (Gb) (S2 Table) from leaves, roots and flower buds of ‘Maillette’. After trimming on quality, length, ambiguous nucleotide and adapters, a total of 144,322,837 reads totaling 19.7 Gb (77% of initial dataset) remained for *de novo* transcriptome assembly with a mean PHRED score per read around 40.

Depending on the genotype, the number of sequenced paired-end DNA reads ranged from 29,985,684 (4,527,838,284 bp) to 145,227,504 (21,929,353,104 bp) (S2 Table). Given that the genome size of *Lavandula angustifolia* is approximately 870 Mb [23], the mean sequencing depth ranged from 5X to 25X. The clone Maillette reaches the highest sequencing depth because it was used for the Unigene-guided DNA assembly described below.

### De novo RNA-seq assembly

Results of *de novo* assemblies are presented on Table 1. Overall, TRINITY generated more contigs (280,062 sequences versus187,584 sequences for CLC assembly) of a longer size than (N50 of 1250bp versus 739 bp for CLC assembly). Likewise, there were almost twice more contigs with a predicted ORF within the TRINITY assembly.

**Table 1:** Assembly metrics for Maillette *de novo* transcriptome assembly with CLC and Trinity tools.

Description	Maillette TRINITY	Maillette CLC
	Assembly metrics	
Number of assembled contigs	280.062	187.584
Minimum contig length (bp)	201	200
Maximum contig length (kp)	15.6	14.6
Assembly size (bp)	220,348,569	109,677,226
Mean contig length (bp)	786.78	584.68
Number of contigs > 1 kb	70.988	25.762
Number of contigs > 10 kb	20	1
Number of contig with predicted ORF (percent)	96,630 (66.20%)	50,399 (72.47%)
N50	1250	739
GC percent	42.84	42.97
Linguistic complexity	0.14942	0.11786
	TRANSRATE quality assessment	
Number of available PE read	66,107,230	66,107,230
Number of mapped PE reads (percent)	62,126,811 (94%)	62,068,704 (94%)
good_mappings (percent of mapped reads)	37,495,608 (60%)	35,932,087 (58%)
potential_bridges	91.104	57.505
Number of base uncovered (percent)	15,251,707 (7%)	507410 (0.5%)
Number of contigs with at least 1 uncovered base (percent)	152,145 (54%)	58,033 (31%)
Contings with a mean per-base coverage <1 (percent)	11,086 (3.95%)	212 (0.11%)
Contings with a mean per-base coverage < 10 (percent)	168,024 (59.99%)	73,318 (39.08%)
Number of contigs putatively segmented (percent)	30,662 (10.95%)	23,851 (12.72%)
Raw assembly score	0.10897	0.15453
Optimal assembly score	0.19831	0.18401
	Completeness assessment with BLASTX alignment	
Total number of optimised contigs	194.706	147.672
Number of contigs with Best Blast Hit (percent)	78,929 (40.53%)	51,821 (35.09%)
Number of unique Best Blast Hit	18.024	16,270
Number of hit with > = 70% coverage	10.508	8.286
Mean number of contigs/Best Blast Hit	4.37	3.18

The TRANSRATE tool used reads data in pairs to assess the quality of assemblies (Table 1). A total of 66,107,704 paired-end reads (92% of cleaned reads) remained in pairs after independent trimming of the reads 1 and the reads 2. More than 62 million of pairs mapped back to the contigs assembled with TRINITY and CLC. Of these mapped paired-end reads, almost 60% were assessed to be mapped in “good” pairs according to the TRANSRATE standards that are: both members of the pair are aligned on the same contig, in the correct orientation and the total length of the reads align against the contig. If the TRANSRATE score obtained for CLC raw assembly (0.154) was better than for TRINITY raw assembly (0.108), the scores were similar for optimized assemblies with a score of 0.184 and 0.198 for CLC and TRINITY assemblies, respectively.

#### Finally, a pool of 147,672 contigs from CLC raw assembly and 194,706 contigs from TRINITY raw assembly were selected as two optimized assemblies for downstream analyses

**Fig 2:**
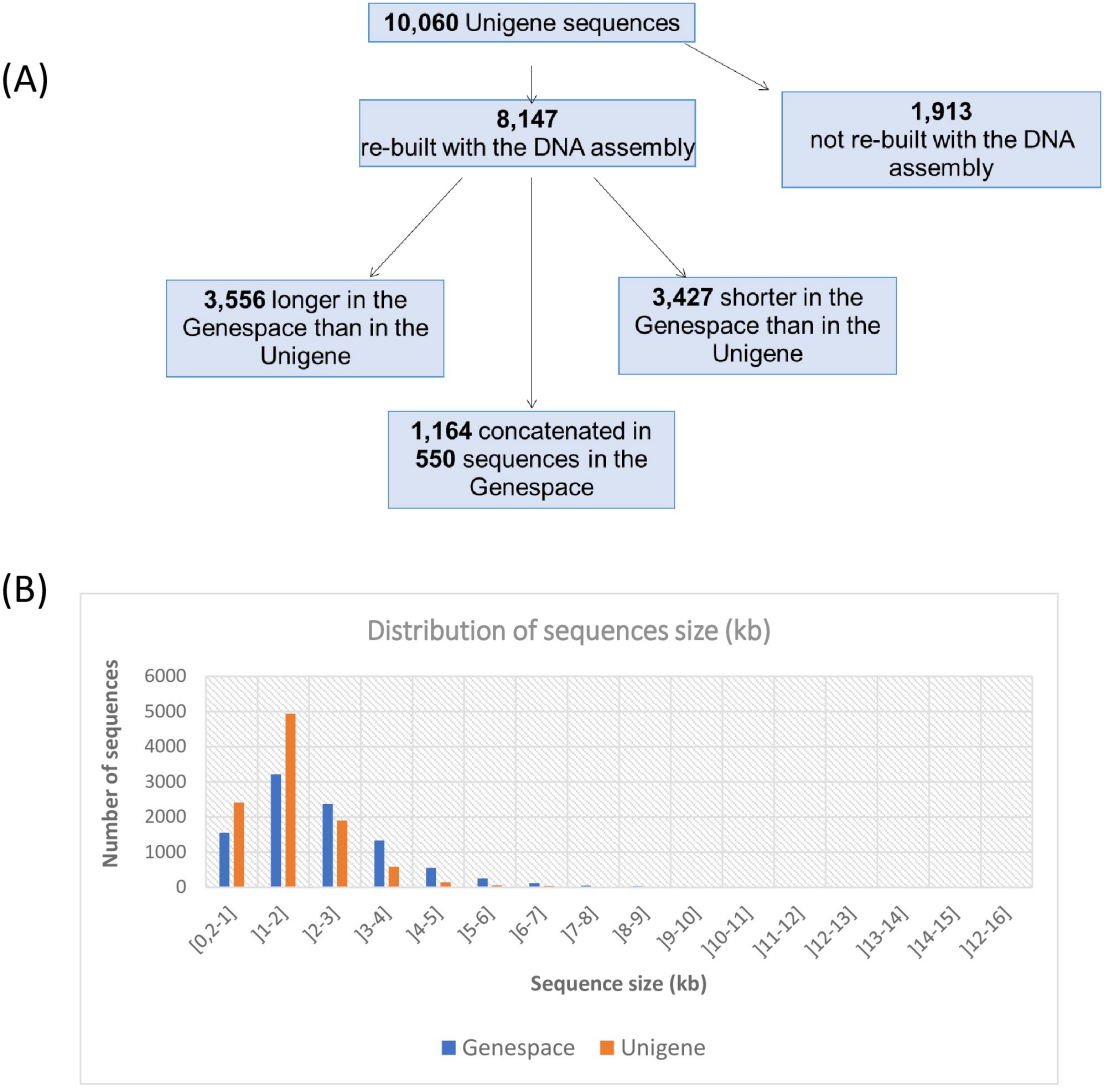
Number and size of sequences built in the Genespace in comparison to the Unigene sequences. (A) A BLASTN alignment was performed to evaluate the number of sequences from the reference Unigene that were recovered in the Genespace. Half of the Unigene sequences were reconstructed and improved in the Genespace (B) Distribution of sequences size in the Genespace and in the Unigene. The Genespace is enriched in sequences of more than 2 kilobases (kb).

To assess the completeness of the optimized assemblies, we used a TRINITY utility (see Methods) to perform a BLASTX alignment against SwissProt database (e-value 1e-20) (Table 1). A total of 51,821 (35%) contigs built with CLC and 78,929 (40.5%) contigs built with TRINITY had a hit in the database. There was redundancy in both optimized assemblies since the 51,821 sequences from CLC optimized assembly matched 16,270 unique proteins and the 78,929 sequences from TRINITY optimized assembly matched 18,024 unique proteins of SwissProt database. This result suggested the presence of homologous sequences such as gene families in the assemblies. **Thus, we took advantage of both assemblies** (see Methods) **and used the BLASTX results to construct a non-redundant reference Unigene of 10,060 contigs**. From those, 8,951 (89%) contigs were deciphered with CLC and TRINITY, while the remaining were only assembled with TRINITY (739 contigs (7%)) or CLC (370 contigs (4%)) (Fig 2A). Selected sequences have length ranging from 202 bp to 15,595 bp with a median value of 1,480.5 bp and a N50 value of 1,913 bp (Fig 2B).

### *In silico* annotation of the reference Unigene

Of the 1440 BUSCO genes, 791 genes (55%) were recovered in the reference Unigene. Among these 791 genes, 729 (92%) were complete and single-copy, 62 genes (7.8%) were complete and duplicated and 23 (0.2%) were fragmented (S2 Fig). The reference Unigene was annotated with various pipelines and softwares to retrieve gene ontology (Trinotate, TRAPID), KEGG pathways (Trinotate, KAAS), gene families (TRAPID) and additional functional annotation (Trinotate pipeline). Detailed results from these softwares are summarized in S3 Table. Of the 10,060 sequence of the reference Unigene, 9,816 (97.6%) had a BLASTP hit against Swissprot database, 8,928 (88.7%) sequences had at least one GO annotation, 4,931(49%) sequences had at least one KEGG pathway annotation and 4,647 (46%) sequences had a KEGG and a GO annotation. Search of conserved domain against Pfam database allowed to increase the rate of sequence annotated with a GO term or a KEGG ontology (S3 Table). The identification of various GO terms and KEGG pathways indicated that we assembled sequences related to a relatively diversified panel of protein functions.

### Introns insertion in the reference Unigene sequences

According to Aluome *et al*. [33], an iterative Unigene-guided DNA assembly was performed to insert introns into Unigene sequences (Fig 1).

Out of the 10,060 sequences of the reference Unigene, 8,147 (81%) sequences were re-built after the 8th iteration of the Unigene-guided DNA assembly from ‘Maillette’ DNA-seq data. A BLASTN alignment of the Unigene sequences against de DNA assembly revealed that, 3,556 (43.64%) Unigene sequences matched longer sequences in the DNA assembly than in the Unigene; 1,164 (14.28%) were concatenated in 550 sequences in the DNA assembly; and 3,427 (42.06%) sequences matched shorter sequences in the DNA assembly.

A total of 1,913 sequences of the Unigene (19%) did not have a hit in the assembled DNA-seq.

**Fig 3:**
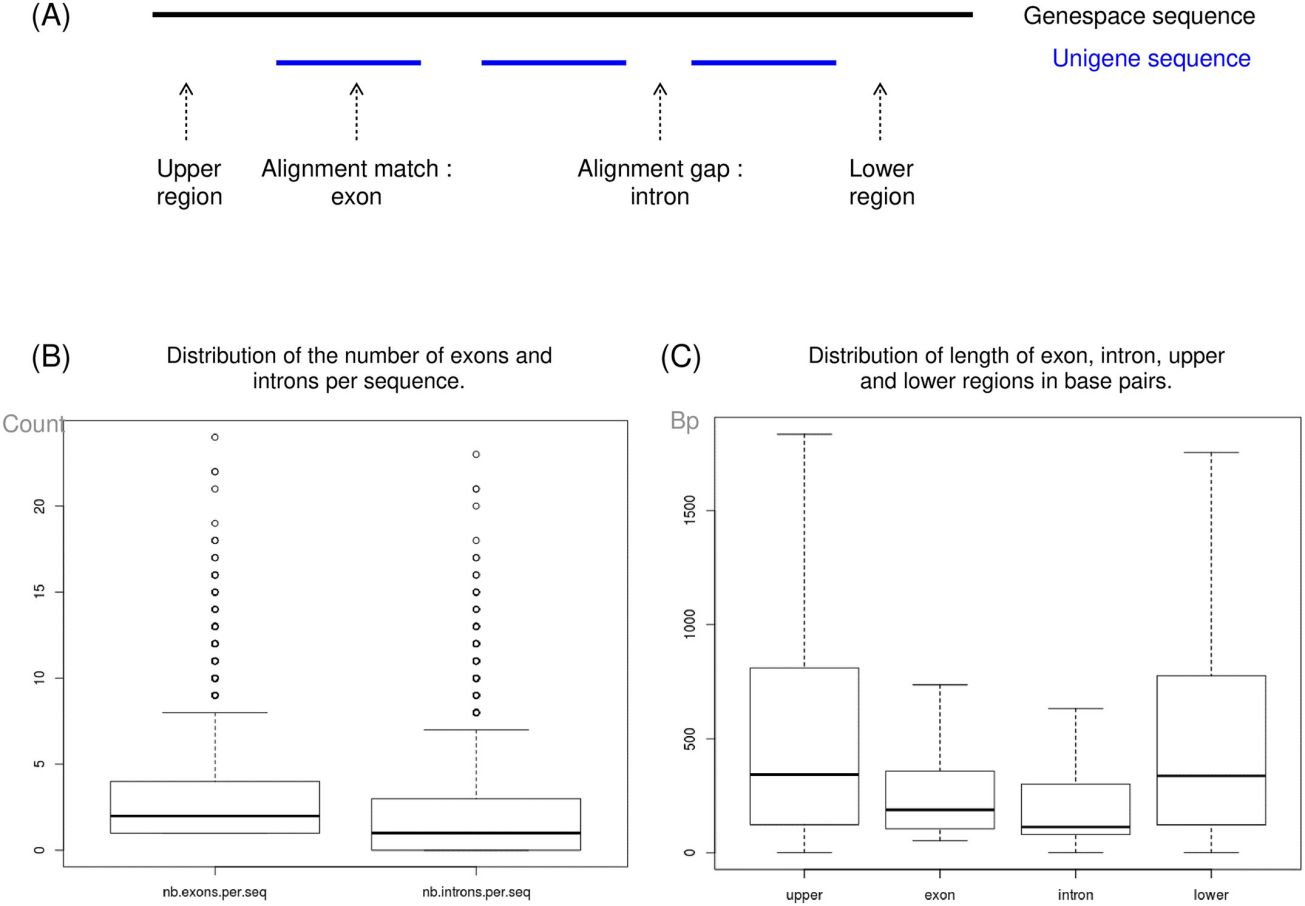
Distribution of exons and introns in the Genespace sequences. (A) **Diagram of the BLASTN alignment of an Unigene sequence against its corresponding Genespace sequence**. Unigene sequence, built from RNA sequencing reads, are mainly compound of exonic regions, whereas Genespace sequences, built from DNA sequencing reads are compound of exonic and non-coding regions. This results in a dashed alignment, indicative of the position, the number and the length of introns and exons on the Genespace sequences. (B) **Boxplot of the distribution of the number of exons and introns per Genespace sequence**. (C) **Boxplot of the distribution of the length (in bp) of exon, intron, upper and lower regions on the Genespace sequences**. Outliers have been removed from the boxplot for a better readability of the graph.

The alignment (BLASTN) of the Unigene contigs against their corresponding gene sequence from the DNA-seq assembly allowed to estimate the number and the length of introns and exons in the gene sequences (Fig 3). Thus, the number of exons per sequence ranged from 1 to 24 with a mean value of 3 (median = 2); and the number of introns per sequence ranged from 0 to 23 with a mean value of 2 (median = 1). In addition, we noticed that the size of the exons and introns was roughly equivalent. Exons size ranged from 54 bp to 4,000 bp (mean = 288 bp, median = 189 bp), and introns size ranged from 0 to 3,800 bp (mean = 235 bp; median = 114 bp). The mean percent of identity for a High-scoring Segment Pair alignment (Fig 3) was of 98%, indicating that, when existing, sequences reconstructed with the DNA-seq assembly were almost identical to the ones built with *de novo* RNA-seq assemblies.

For SNP detection, we built a reference set called "Genespace" composed of all the sequences initially present in the Unigene but in their longest form either coming from the de novo assembly of RNA-seq, or from the DNA-seq assembly, considering the assumption that the longer the sequence, the larger the information retained. The 1,913 sequences that were not reconstructed with the DNA assembly, but which were in the Unigene were also included in case their missingness was only due to assembly issues during the iterative DNA assembly. **The Genespace resulted in a set of 9,446 sequences (21.4 Mb)**.

### SNP detection

The first step consisted in conducting 16 individual mapping jobs with the DNA sequencing reads from the 16 clones. For each mapping job, almost 6% of the DNA-seq mapped on 85% (more than 8000 sequences) of the Genespace sequences and, at least 90% of the length of each reference sequences was covered by one or more DNA sequencing reads. A total of 1,442 Genespace sequences (1.3Mb– 6% of the Genespace size) were not successfully mapped with DNA-seq reads in none of the 16 mapping jobs. These sequences were part of the 1,913 Unigene sequences that were not rebuilt with the DNA-seq assembly (see previous section). Further investigation indicated that these sequences corresponded to sequences from micro-organisms known to be part of plants rhizosphere (these sequences are identified in the 2^nd^ column of S3 Table).

Primary SNP discovery was performed independently with CLC and GATK. The selection of the subset of concordant SNP between the two tools (position + genotype calling) led to the construction of a genotyping matrix of 16 individuals * 359,323 sites (S4 Table). These SNP spread on 7,332 Genespace sequences with a mean value of 1 SNP per 90 bp. The average depth of coverage of an SNP in the panel of 16 individuals is between 0.25X and 50X (median = 7.8X) (S3A Fig). Each polymorphic site being supported by 5 to 800 reads summed across the 16 clones (S3B Fig). The minimum value of 5X was tolerated when a polymorphic site reached enough quality to be called, in only one of the 16 individuals. Interestingly, 114 to 1,281 putative private polymorphisms (*i*.*e*. allele found in one clone but not the others) were found within the lavender clones, with 20 to 121 SNP being at homozygous state (S4 Table). Moreover, 30,144 putative private SNP including 991 SNP at the homozygous state were identified between the lavandin ‘Grosso’ and all the lavender clones.

For downstream analyses, we used a subset of 9, 505 SNP generating no missing data across the 16 individuals (see vcf file in S5 Table). Of these 9505 SNP, 66% had a minor allele frequency (MAF) ranging from 0.4 to 0.5, 13.5% had a MAF value in the range 0.05–0.4 and 20.5% had a MAF value inferior or equal to 0.05. Rare alleles were deliberately preserved in the dataset to maximize our power to discriminate among the individuals. The majority (98.7%) of the conserved SNP were biallelic (S6 Table). The genotype of the 16 clones at these 9,505 SNP was mainly heterozygous with one reference and one alternate allele (66.7 to 80.9% of the genotype calls, S6 Table).

### Genetic distances analysis

**Fig 4:**
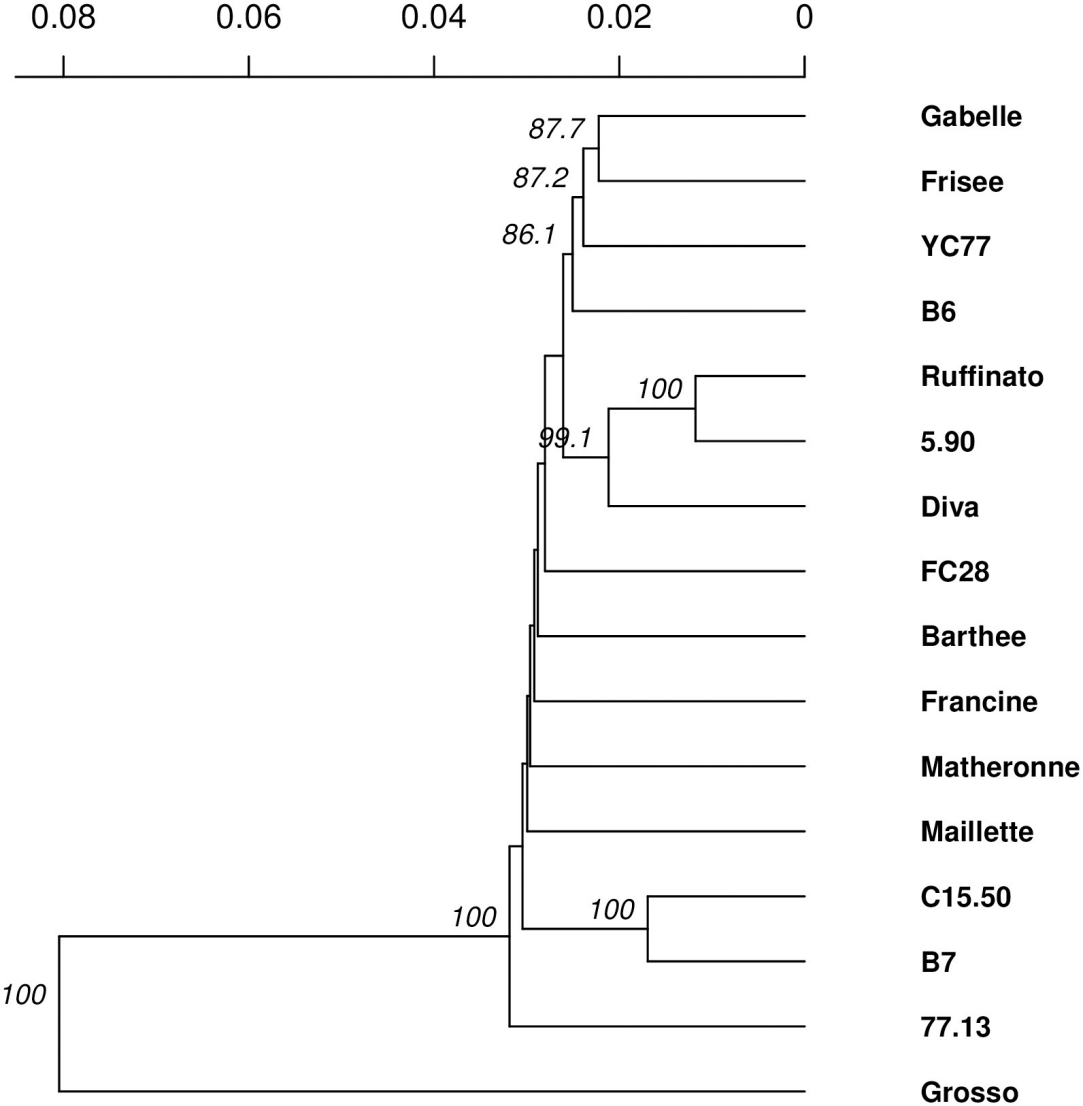
Phylogenetic tree of the studied lavenders and lavandin. Built from a distance matrix calculated from 9,505 SNPs, this UPGMA tree suggests 3 large groups in our panel, within which some clusters are well supported by the bootstrap values.

Pairwise genetic distances calculated from the 9,505 SNP ranged from 0.023 (Ruffinato/5.90 pair) to 0.167 (77.13/Grosso pair) (S7 Table). Most of the clones are genetically close: 75% of the calculated distance pairs were between 0.05 and 0.07. However, all the clones were successfully discriminated with the set of SNPs. As expected, the clone ‘Grosso’ appeared to be divergent from the lavender clones with a mean genetic distance to other lavenders of 0.16. The distance matrix was used to perform a UPGMA analysis with 1000 bootstraps (Fig 4). Three main cluster seems to be highlighted: C15.50/B7/Barthée, Gabelle/Frisée (the two “blue lavender” of the dataset), and Maillette/Diva/77.13/Ruffinato/5.90.

**Fig 5:**
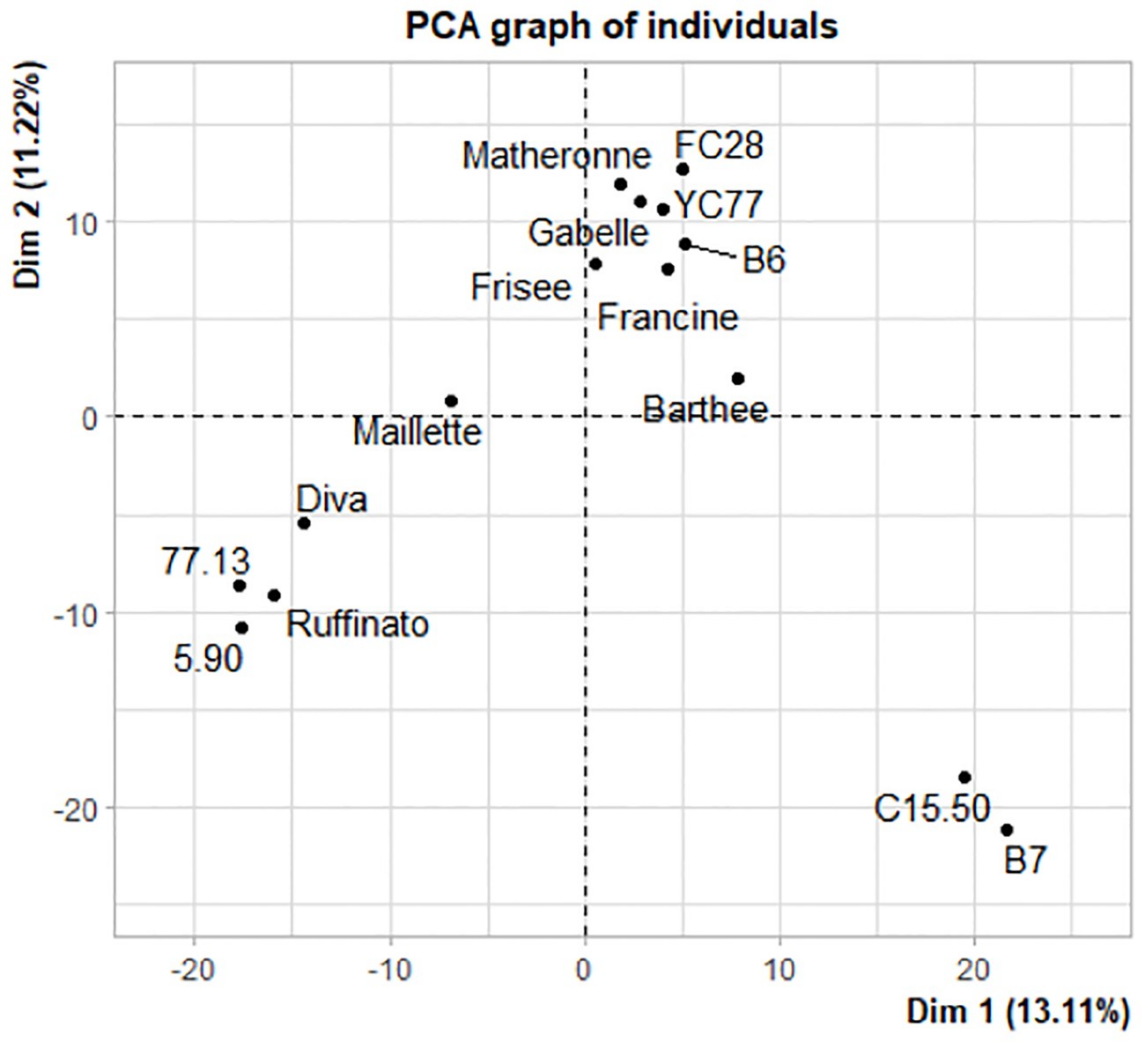
Principal component analysis of the genetic distances between the 15 lavenders. The first two axes explained 13.07% and 11.22% of the variability observed between these clones.

To better assess the intra-species diversity, a PCA was performed between the lavenders (Fig 5). The first three axis explained 13.07%, 11.22% and 9.27%, respectively, of the variability observed between these clones. Overall, the analysis highlighted the same clusters as the UPGMA analysis. The two clusters compound of C15.50/B7 on one side, and 5.90/Ruffinato explained most of the variability along the first axe. Likewise, the cluster with the clones C15.50/B7 on one side and B6/Francine/Matheronne, FC28/YC77/Frisée/Gabelle mainly explain the variability along the second PCA axe.

## Discussion

In this study, we proposed a methodology for the rapid development of large-scale molecular resources for a species with limited available genomic information. We studied as an example *Lavandula angustifolia* Mill., a species of great economic importance from the *Lamiaceae* family. Our goal was, firstly to develop genomic reference sequences using RNA and DNA sequencing data. Secondly, to use these resources for SNP mining and finally tested the SNP within the scope of a genetic distance analysis of lavender clones. In this section, we discuss the efficiency, the advantages, and the possible improvement of the method.

### Construction of a good-quality gene sequences using RNA and DNA sequencing data

We built gene sequences from 84.5 million of RNA paired-end reads and 145 million of DNA paired-end reads sequenced from leaves, roots, and flower buds of the lavender clone ‘Maillette’. We used different licensed and free softwares for our analyses, however this can be done with any of one’s preferred tool. The bioinformatic platforms provide users with suite of tools and pipelines to make and validate a *de novo* assembly of RNA-seq such as DRAP [45] or the Trinity protocol [26]. Likewise, the transcriptome-guided DNA-seq assembly protocol can be performed with any tool allowing to map reads to a reference genome and then extract reads that mapped in proper and broken pairs.

#### De novo transcriptome assembly

The quality of an assembly is primary based on the quality and the quantity of sequencing reads. In light of previous studies, in which an amount of 20–40 million of reads originating from various tissues were recommended to get a comprehensive assembly [46], we had enough data to get relevant sequences from RNA data. To assess the assembly quality, it has been demonstrated that the best metrics included (i) the proportion of reads mapping back to the assembly, (ii) the recovery of conserved, widely expressed genes and (iii) the total number of Unigene sequences [46,47].

In this study, we used the tool TRANSRATE to calculate the proportion of reads that mapped back to the *de novo* assembled contigs. Results indicated that 94% of read pairs mapped to the contigs but this value dropped to ~60% when considering reads that mapped in a “good manner” as defined by Smit-Unna *et al* [27]. It is difficult to compare this value since this metric is not yet widely used. Similar studies only provide the global percent of reads mapping back to the reads [6,46–51]–which, in our case, indicate that the assemblies are well supported by read data.

To assess the recovery of genes, we chose to perform a BLASTX alignment analysis against the SwissProt database and finally selected sequences that represent nearly full-length proteins to make our reference Genespace, that is contigs that cover more than 70% protein-length of a homologous protein from SwissProt database. With these supplementary selective conditions, we restricted ourselves to a narrow annotatability of the reference Unigene and we may have missed out some well assembled sequences or biased our reference Unigene towards well-conserved sequences between species. However, according to our goal that was to develop molecular resources with a high level of reliability, this choice was appropriate to prevent from keeping sequences with assembly errors [52,24]. Despite filtering steps and due to the database selected for BLASTX alignments, well-assembled contaminant sequences were identified in the 10,060 sequences firstly selected in the Unigene and were subsequently removed to **obtain a cleaned reference set of 8, 640 coding sequences**.

The BUSCO results indicated that our cleaned reference Unigene is partly complete. Indeed, the level of completeness of our Unigene reached 55% of the BUSCO Embryophyta data set. This result is lower than that obtained from the whole genome sequencing of lavender for which 92% of completeness was achieved [23]. However, our reference is of good quality because most of genes found are complete and in single copy. Only 8% of genes are fragmented or duplicated [28]. Moreover, the number of complete genes in single copy is higher in our Unigene (729 genes) compared to the complete genome (592 gene). **Thus, this Unigene provides complementary information with respect to the complete genome**. Lastly, gathering the results obtained from two or more assemblies allow to reduce the number of sequences due to bioinformatics artefacts and thus, to increase the assembly quality [24]. In our reference Unigene, almost 90% of selected sequences where assembled with both Trinity and CLC indicating the high-quality and reliability of sequences assembled herein.

#### Introns insertion

To perform a good SNP detection, the first step consisting in the alignment of sequencing reads from individuals on a reference sequence is of great importance. The originality of this study lies on the insertion of intronic sequences within the exons of the Unigene sequences to recover full-length gene sequences. This has the advantage to improve read mapping quality at exon/intron junctions and to increase the number of reads mapping on the reference sequences, the two resulting in increasing the putative number of SNP detected. In addition, there is potentially more polymorphism in the introns than in the exons because these regions are expected to be less subject to selection pressure. Thus, by adding introns, we maximized the possibility of finding polymorphism between genetically close individuals.

In this study, DNA assembly for intron insertion allowed the reconstruction of 81% of the sequences initially present in the reference Unigene versus 88% in the study of Aluome *et al*. [33] in which this method was developed. Among these reconstructed sequences, we found that the size of some sequences was shorter in the Genespace than in the Unigene. This might be due to assembly issues. One hypothesis would be related to sequencing depth and the high polymorphism frequency in the clone used in this study. Indeed, since the sequencing depth reached in the RNA-seq was larger than in the DNA-seq, some complex sequences may have been built in the Unigene but not in the Genespace due to a lower sequencing depth leading to assembly premature stops at complex regions.

We obtained Genespace sequences that had on average 3 exons and 2 introns. This result is in concordance with mean values observed in plants. Moreover, the higher amount of exons versus introns was also observed in *L*. *angustifolia* whole genome analysis [23]. Likewise, the size of intronic and exonic regions is in the order of magnitude of what is observed in perennial plants such as *Arabidospsis lyrata* [53], *Fragaria vesca* [54] or trees [55].

### SNP discovery and genetic distances analysis

SNP detection from 16 lavender lavandin clones allowed to discover 359K putative SNP, with a high SNP frequency (mean of 1 SNP per 90 bp) and a high level of heterozygosity (up to 60% of heterozygous SNP genotype/individual). This result is consistent with the outbreeder nature of lavender and the breeding scheme applied that is based on massal selection with clonal propagation. In the study of Adal *et*.*al* [22], where SSR markers were developed for lavender, authors also concluded a high polymorphism frequency (1 SSR/2.1kb) in comparison to model crops such as rice (1 SSR/3.4 kb), wheat (1 SSR/5.4 kb) or soybean (1 SSR/7.4 kb)). Similar results have been reported for other outbreeder perennial plants with recent domestication history such as ryegrass [56].

This study is the first one to report genetic distance analysis at the molecular level between many cultivated clonal lavender varieties with SNPs. The main conclusions that can be drawn from these results are: (i) on overall, the homogeneous genetic distances between pairs of clones, related to the allogamous nature of the species, a restricted area of cultivation and the varietal creation method; (ii) as well as an absence of structuring related to the geographical origin. This last result must be moderated because we had only two Bulgarian clones in the study. However, it could corroborate the hypothesis stating that couples of years ago, there were plant material exchanges across countries (CRIEPPAM, personal communication), resulting in a shared genetic origin between those different cultivars. This result also suggests the need of searching for structuring factors across lavender cultivars other than geographical origin. One clue given by our analysis is the agronomical characteristics. Indeed, the cluster compound of Gabelle and Frisée can be explained by the fact that these two are "blue" lavender, selected for the persistence of the flowers after the cut, whereas all the other lavenders studied in our collection had been selected for their quality and/or their yield of essential oil. Similarly, from a phenotype point of view, the Diva and Maillette clones share similar agronomical characteristics, even if they have distinguished chemotypes (CRIEPPAM, personal communication).

Our results are corroborated with those found in the literature. The low genetic differentiation within *L*. *angustifolia* observed in this study have been previously reported [28]. In a study including wild and improved lavender populations, blue lavenders and clonal varieties—some of which were included in our collection, Chaisse *et al*. [57] also reported the genetic proximity between C15.50 and B7 as well as a notable structuring of the diversity between the blue lavenders and the clonal varieties selected for their essential oil content. The results presented here are as many clues to decipher the structuring of genetic diversity within the species and to trace the history of the domestication of *L*. *angustifolia* and its related species. A larger scale analysis including most of the genetic resources available will help achieving this task.

## Conclusion

This study presents an original way to use RNA-seq and DNA-seq assemblies to develop molecular resources on a species for which no genomic information is available. This method has the advantage of allowing the detection of SNPs in intronic regions, that are expected to exhibit more variability than exons, and thus is also suitable to self-pollinated species or genetically close individuals. The results presented in this study are complementary to those published on lavender since our data do not target the terpene biosynthesis pathway that had been mainly studied on lavender, and thus the data is available for a wider range of applications. Moreover, this is the first reported large-scale SNP discovery on *Lavandula angustifolia*. In regard with the low genetic differentiation between cultivars, high resolution molecular markers such as SNP would be required to accurately explore the genetic diversity of lavender.

All this data provides a rich pool of molecular resource to initiate genomic approaches in lavender research.

## Supporting information

S1 Fig Pipeline for the construction of the reference Unigene. (PDF)

S2 Fig Comparison of the completeness of lavender Unigene with the completeness of *Lavandula angustifolia* and *Mentha longifolia* genomes using BUSCO. (PDF)

S3 Fig Distribution of depth of coverage (mean and total coverage) of the 359K SNPs selected. (PDF)

S1 Table List of lavender clones studied. (PDF)

S2 Table RNA and DNA sequencing metrics. (PDF)

S3 Table Reference Unigene annotation results with Trinotate, Trapid and KAAS tools. (XLSX)

S4 Table Detailed results of SNP detection with GATK and CLC (concordant SNP only) within the 16 clones. (XLSX)

S5 Table Variant Calling File of the 9,505 SNP generating no missing data across the 16 lavenders clones. (VCF)

S6 Table Distribution of genotype calls at the 9,505 SNP generating no missing data across the 16 individuals. (XLSX)

S7 Table Distribution of pairwise genetic distances. A set of 9,505 SNPs generating no missing data were used to calculated pairwise genetic distances with Provesti’s method. All the lavenders had pairwise genetic distances ranging from 0.02 to 0.08. The lavandin Grosso had genetic distances with the lavenders comprised between 0.15 and 0.17.
(XLSX)

S1 File Bash script used for introns insertion in Unigene sequences. (SH)
