# Supplementary figures and images for: Quick and efficient approach to develop genomic resources in orphan species: Application in Lavandula angustifolia
Source: PLoS One. 2020 Dec 11;15(12):e0243853. doi: 10.1371/journal.pone.0243853 (PMC7732122; doi:10.1371/journal.pone.0243853)

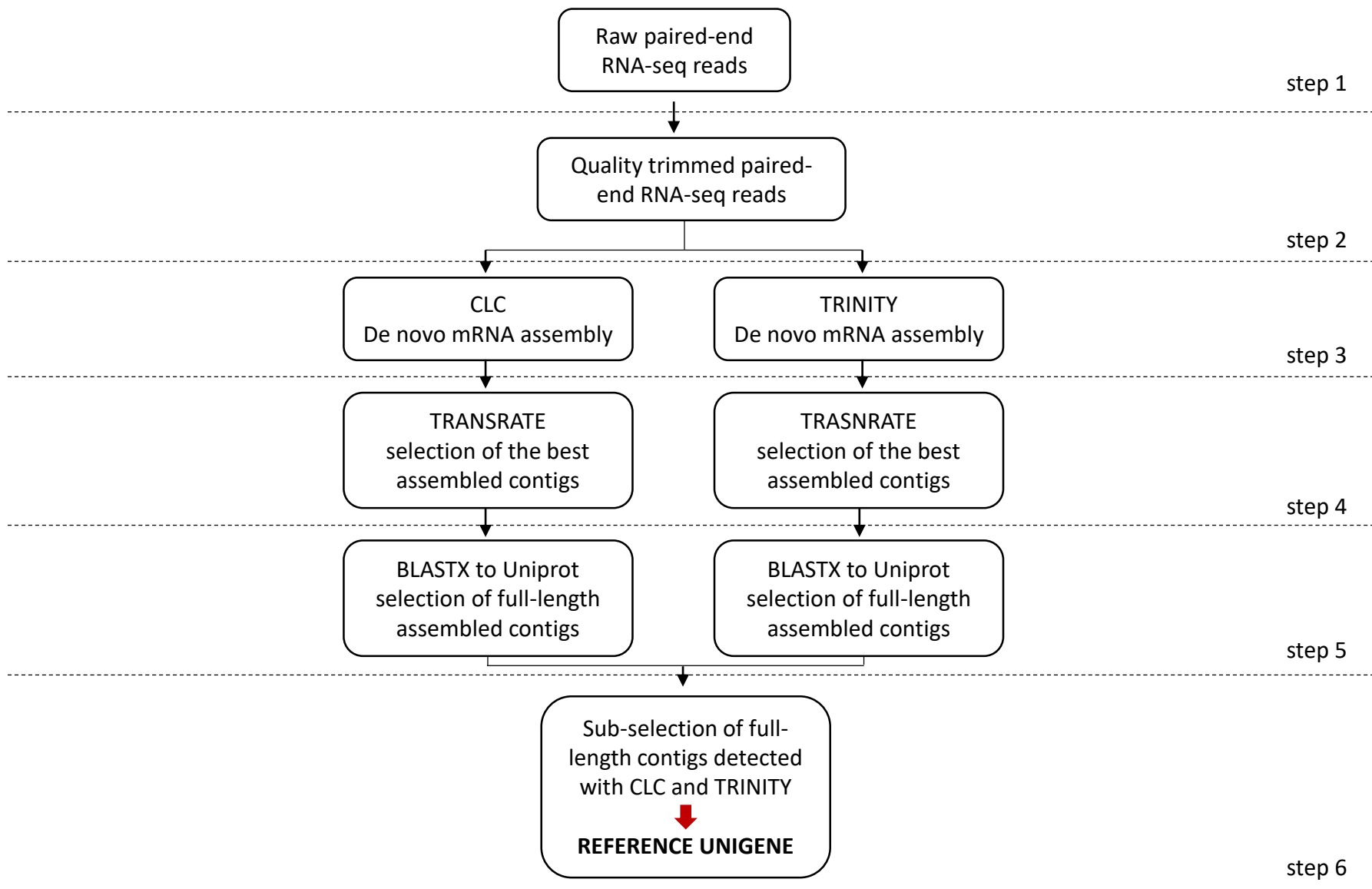

Supplement: S1 Fig — (PDF) [file pone.0243853.s001.pdf]
