# Supplementary material for: Quick and efficient approach to develop genomic resources in orphan species: Application in Lavandula angustifolia
Source: PLoS One. 2020 Dec 11;15(12):e0243853. doi: 10.1371/journal.pone.0243853 (PMC7732122; doi:10.1371/journal.pone.0243853)
Supplement: S2 Fig — (PDF) [file pone.0243853.s002.pdf]

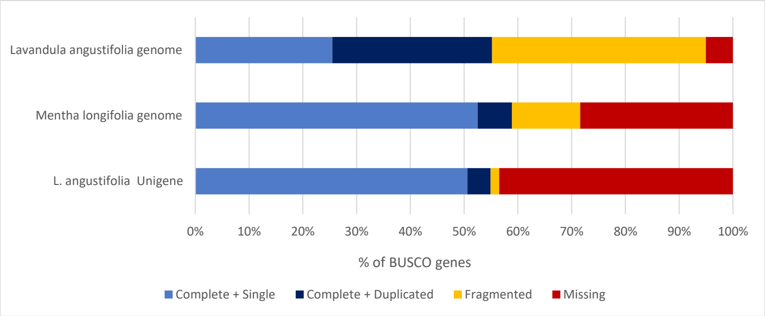

S2 Fig. Comparison of the completeness of lavender Unigene with the completeness of *Lavandula angustifolia* and *Mentha longifolia* genomes using BUSCO.
