# Supplementary material for: Quick and efficient approach to develop genomic resources in orphan species: Application in Lavandula angustifolia
Source: PLoS One. 2020 Dec 11;15(12):e0243853. doi: 10.1371/journal.pone.0243853 (PMC7732122; doi:10.1371/journal.pone.0243853)
Supplement: S3 Fig — (PDF) [file pone.0243853.s003.pdf]

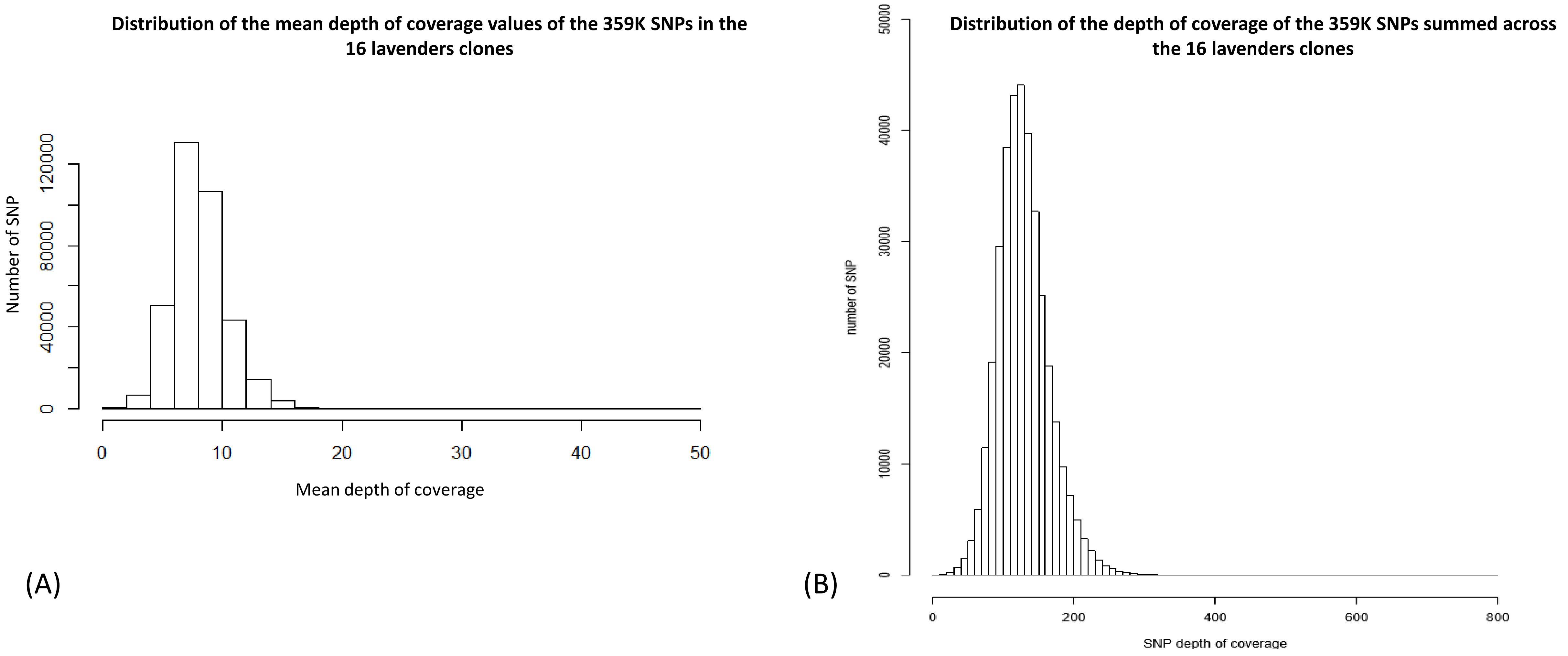

S7 Fig. Results for SNP detection in 16 lavender clones.

(A) Distribution of the mean depth of coverage of SNP in the 16 lavender clones. Data presented for the 359K SNP.

(B) Distribution of the depth of coverage summed across the 16 lavender clones. Data presented for the 359K SNP.
