# Supplementary material for: Quick and efficient approach to develop genomic resources in orphan species: Application in Lavandula angustifolia
Source: PLoS One. 2020 Dec 11;15(12):e0243853. doi: 10.1371/journal.pone.0243853 (PMC7732122; doi:10.1371/journal.pone.0243853)
Supplement: S1 Table — (PDF) [file pone.0243853.s004.pdf]

Cultivars	Geographical origin	Detailed geographical origin	Variety origin	Selection criterion	Comments
Maillette	France	Drôme	Farmer breeding 40's-50's.	Essential oil quality, disease resistant and easy to grow in plains. High yield	Widely planted since the 50's. At least 5 clones of Maillette grown il the Alpes-de-Haute-Provence (McLeod, 2000).
5.90	France	South-east		Essential oil yield and quality	
77.13	France	Plateau d'Albion (Sault)	Collected from a field of open-pollinated variety late 90's.	Essential oil yield and quality. Tolerant to yellow decline	Commercialized in early 2000.
B6	Bulgary	NA	Cultivar obtained from bulgarian breeding program.	Essential oil yield and quality	Suceptible to yellow decline. Referred to as "blue lavender" in an activity report of CRIEPPAM (M. Pellissier, pers. Com.).
B7	Bulgary	Kasanlak	Cultivar obtained from bulgarian breeding program.	Essential oil yield and quality	Suceptible to yellow decline.
Barthée	France	Plateau d'Albion (Sault)		Essential oil yield and quality	Similar to Matheronne -agronommical characteristics- only larger in size.
C15.50	France	Plateau d'Albion	Collected from a field of open-pollinated variety.	Essential oil yield and quality. Initially tolerant to yellow decline	Quite suceptible to yellow decline nowadays. Commercialized since 90's.
Diva	France	Plateau d'Albion	Collected from a field of open-pollinated variety.	Essential oil yield and quality. Tolerant to yellow decline. High yield.	Production level equivalent to Maillette.
FC28	France	Plateau d'Albion	Collected from a field of open-pollinated variety.	Essential oil yield and quality	Commercialized since 90's.
Francine	France	Drôme (Le Pègue)	Collected by the Chambre d'Agriculture de la Drôme before 1989.	Essential oil yield and quality	
Frisée	France	South-east		Cut flower	
Gabelle	France	South-east		Cut flower	
Matheronne	France	South-east	Farmer breedin in the 40's-50's	High yield of very good quality essential oil. Tolerant to yellow decline	Seems to share a common ancestry with at least 77.13, C15.50 and B7 (Chaisse et al., 2012).
Ruffinato	France	Plateau d'Albion	Collected from a field of open-pollinated variety.		Bad essential oil quality.
YC77	France	Plateau d'Albion	Collected from a field of open-pollinated variety.		
Grosso	France	Vaucluse	Spontaneous hybrid discoverd in 1972. Commercialized in early 80's (Upson et al., 2004).	Essential oil yield and quality	

References

Chaisse E, Foissac X, Verdin E, Nicolè F, Bouverat-Bernier JP, Jagoueix-Eveillard S, et al. Amélioration des stratégies de lutte contre le dépérissement de la lavande et du lavandin. Innov Agron. 2012;25: 179–192.
McLeod, J.A. (2000). Lavender, sweet lavender. (edn 2 revsed) 146 pp. Kangaroo Press, Australia
Upson T. & Andrews S. (2004). The Genus Lavandula 442 pp. Royal Botanic Gardens Kew, UK
