# Supplementary material for: Quick and efficient approach to develop genomic resources in orphan species: Application in Lavandula angustifolia
Source: PLoS One. 2020 Dec 11;15(12):e0243853. doi: 10.1371/journal.pone.0243853 (PMC7732122; doi:10.1371/journal.pone.0243853)
Supplement: S2 Table — (PDF) [file pone.0243853.s005.pdf]

Genotype name	Nb reads sequenced	Nb of bases sequenced	Approximate depth of coverage
RNA seq			
Maillette Leave	53 266 404	8 043 227 004	
Maillette Roots (reads 1)	59 341 540	8 960 572 540	
Maillette Flower buds (reads 1)	56 474 946	8 527 716 846	
**Total**	**169 082 890**	**25 531 516 390**	
DNA-seq- leaves sample			
Maillette	145 227 504	21 929 353 104	25
Diva	65 151 226	9 837 835 126	11
Ruffinato	67 452 366	10 185 307 266	12
B6	89 105 430	13 454 919 930	15
B7	30 768 088	4 645 981 288	5
Gabelle	57 202 964	8 637 647 564	10
Matheronne	29 985 684	4 527 838 284	5
Barthée	33 306 066	5 029 215 966	6
C15.50	106 240 042	16 042 246 342	18
YC77	63 807 640	9 634 953 640	11
Frisée	53 330 332	8 052 880 132	9
5.90	75 616 676	11 418 118 076	13
FC28	36 269 606	5 476 710 506	6
77.13	33 625 350	5 077 427 850	6
Francine	30 954 844	4 674 181 444	5
